# Selenonium Polyelectrolyte Synthesis through Post-Polymerization Modifications of Poly (Glycidyl Methacrylate) Scaffolds

**DOI:** 10.3390/polym12112685

**Published:** 2020-11-13

**Authors:** Taejun Eom, Anzar Khan

**Affiliations:** Department of Chemical and Biological Engineering, Korea University, 145 Anam-Ro, Seongbuk-Gu, Seoul 02841, Korea; eomtorr@korea.ac.kr

**Keywords:** selenium-epoxy reaction, polyselenonium salts, organoselenium polymers, epoxide ring-opening reaction, poly(glycidyl methacrylate)s

## Abstract

Atom transfer radical polymerization of glycidyl methacrylate monomer with poly(ethylene glycol)-based macroinitiators leads to the formation of reactive block copolymers. The epoxide side-chains of these polymers can be subjected to a regiospecific base-catalyzed nucleophilic ring-opening reaction with benzeneselenol under ambient conditions. The ß-hydroxy selenide linkages thus formed can be alkylated to access polyselenonium salts. ^77^Se-NMR indicates the formation of diastereomers upon alkylation. In such a manner, sequential post-polymerization modifications of poly(glycidyl methacrylate) scaffolds via selenium-epoxy and selenoether alkylation reactions furnish practical access to poly(ethylene glycol)-based cationic organoselenium copolymers.

## 1. Introduction

Cationic polyelectrolytes find various bio-relevant applications due to their ability to interact with oppositely charged biomolecules. Typically, ammonium, sulfonium, and phosphonium groups are used to endow the polymer chain with a positive charge. We have recently shown that selenium atoms appended to a polymer chain can be transformed into a cation to afford the first known family of selenonium polyelectrolytes [1]. The synthesis was carried out through a selenium-epoxy reaction [2]. In this reaction, diselenide precursors (R-Se-Se-R) were reduced in-situ with the help of sodium borohydride to generate selonolate (R-Se^–^) nucleophiles. Such nucleophiles can open the epoxide rings to produce ß-hydroxy selenide linkages [3,4,5,6,7,8,9,10,11,12]. A subsequent alkylation of the produced seleno-ethers then affords the side-chain selenonium cations. The reaction conditions, however, can be considered as relatively harsh since sodium borohydride is a strong reducing agent. Therefore, in this work we investigate milder conditions to achieve the synthesis of organoselenonium polyelectrolytes. In addition, new polymer compositions (for instance, copolymer architectures containing poly(ethylene glycol)s) are sought to establish the generality of the concept and to enhance the repertoire of the newly introduced polycation family. The described polymers provide an alternative to the known families of polycations (e.g., ammonium, phosphonium, and sulfonium polymers) and are anticipated to be useful in a range of biological studies [13].

## 2. Results and Discussion

**Scheme 1:**
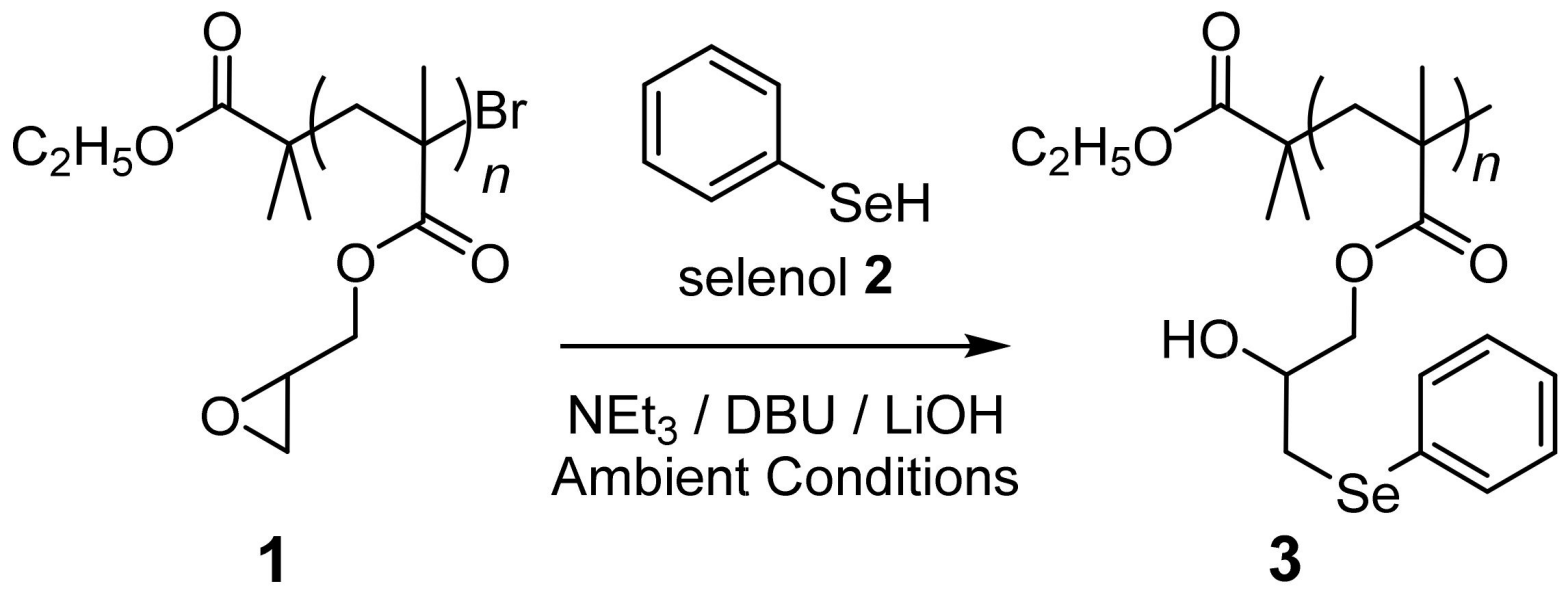
Base-catalyzed ring-opening of side-chain epoxides in poly(glycidyl methacrylate)s through selenium-epoxy reaction.

Selenols (RSeH) possess a relatively low p*K*_a_ (5.2) [14]. Therefore, catalytic amounts of bases such as triethylamine (TEA), diazabicycloundecene (DBU), or lithium hydroxide (LiOH) can form the required selenolate anion for the ring-opening reaction [2]. However, so far, only a small molecule study has been reported in this context [2]. Therefore, initially, an optimization study was carried out using atom transfer radically prepared [15] poly(glycidyl methacrylate) homopolymer **1** (*M*_w(GPC)_ = 41,900, *M*_n(GPC)_ = 36,400, *M*_w_/*M*_n_ = 1.1) and benzeneselenol **2** (Scheme 1) [16].

**Figure 1:**
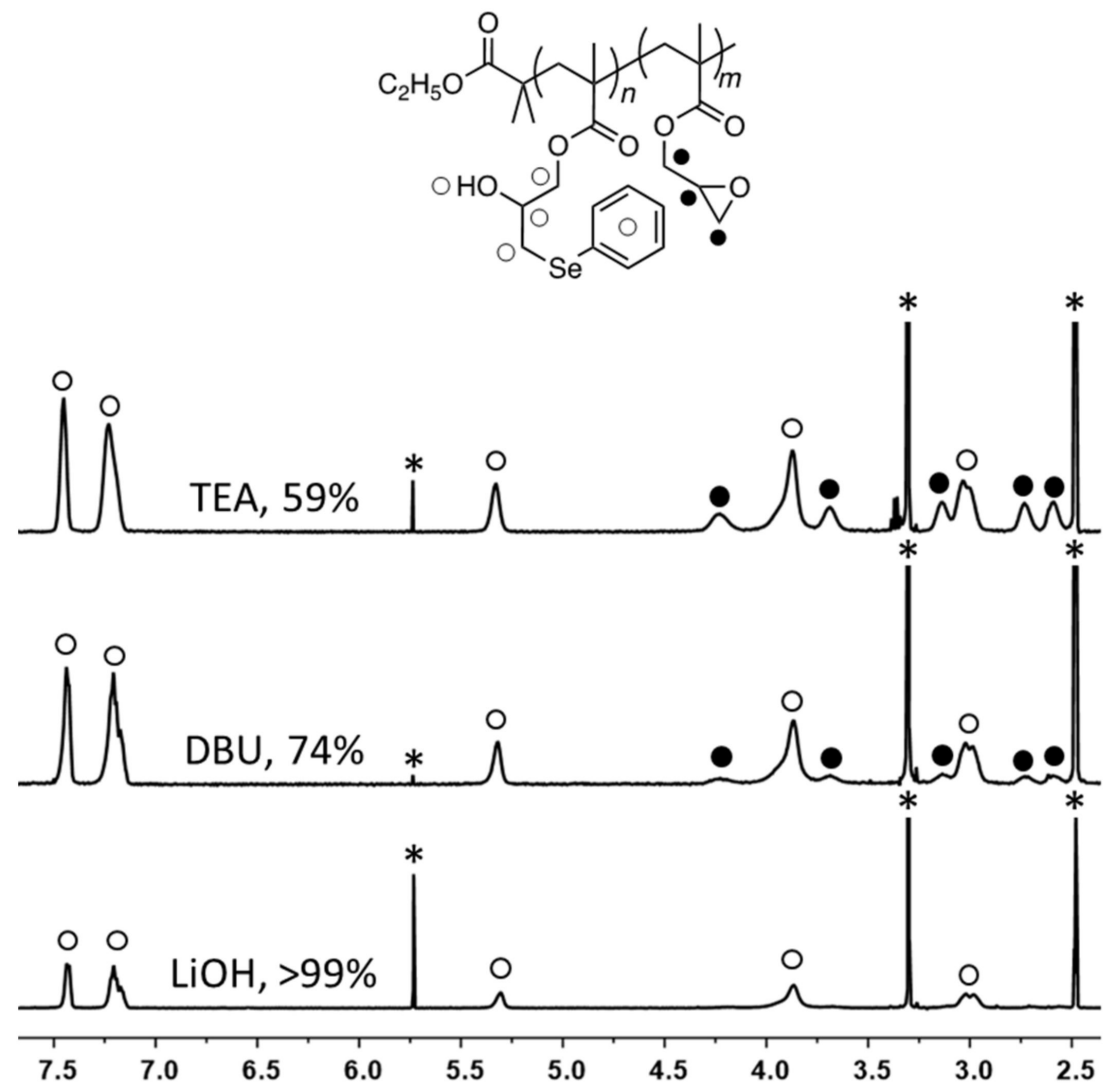
^1^H-NMR (DMSO-*d_6_*) of **1** after ring-opening reaction. The nature of the base and the extent of ring-opening reaction are shown with each data trace. The residual solvent signals from DMSO and water are shown with the help of an asterisk.

**Figure 2:**
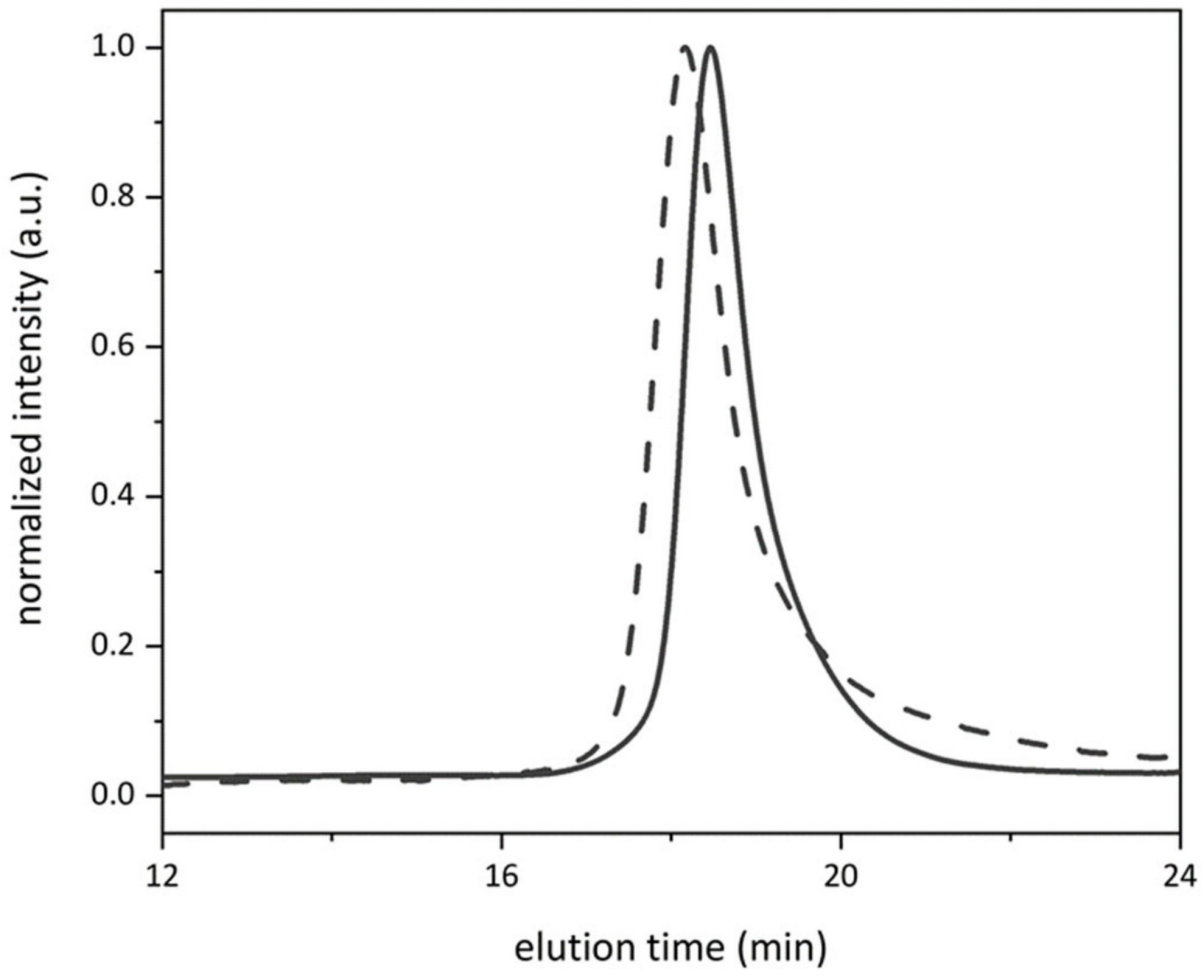
Gel permeation chromatography (GPC) chromatograms of **1** (line) and **3** (dash) in tetrahydrofuran (THF).

In this post-polymerization functionalization [17,18,19,20,21,22] study, the proton resonances from the epoxide group (labeled with filled circles in Figure 1, Figures S1–S8) are followed as a function of reaction time, reaction medium, and the nature and amount of a base to fathom the extent of the ring-opening reaction [23,24,25]. Initially, the reactions were carried out for 12 h with 3.6 mol% of catalyst. In addition, since oxidative dimerization of benzeneselenol to diphenyldiselenide (Ph-Se-Se-Ph) under ambient conditions is a known side-reaction [2], an excess of **2** (1.25 eq./epoxide) was used. Under these conditions, in tetrahydrofuran (THF) and chloroform, the extent of ring-opening reaction remained unsatisfactory (65–84%, Table 1). Only in aqueous THF, LiOH led to a quantitative ring-opening reaction. Therefore, the amount of the catalyst was increased to 5.9 mol%. In these cases, DBU and LiOH both produced a quantitative ring-opening reaction in THF. In chloroform, however, DBU and TEA only resulted in a 91% ring-opening reaction. LiOH worked equally well in both solvent systems, resulting in the quantitative transformation of the side-chain epoxides to the seleno-ethers. Finally, the reaction time was reduced to 3 and 1 h. These reactions indicated that 1 h of reaction time was insufficient for all systems and provided only a 55–86% ring-opening reaction. The reaction time of 3 h, however, was sufficient for LiOH to provide a quantitative reaction in both solvent systems. Therefore, LiOH can be considered as the best catalyst that can be used in chloroform or aqueous THF in a loading of 5.9 mol% to furnish **3** (*M*_w(GPC)_ = 52,400, *M*_n(GPC)_ = 42,500, *M*_w_/*M*_n_ = 1.2) in 3 h. DBU can be used as an alternative in 5.9 mol% loadings to furnish **3** in THF in 12 h of reaction time. Gel permeation chromatography (GPC) indicated that the functionalization reactions did not alter the polymer dispersity in a significant manner, and that the hydrodynamic volume increased due to the addition of the phenyl ring to the polymer side-chain (Figure 2). Based on these results, 5.9 mol% of LiOH in aqueous THF was chosen along with the reaction time of 3 h for future ring-opening reactions on the planned block copolymer scaffolds.

**Figure 3:**
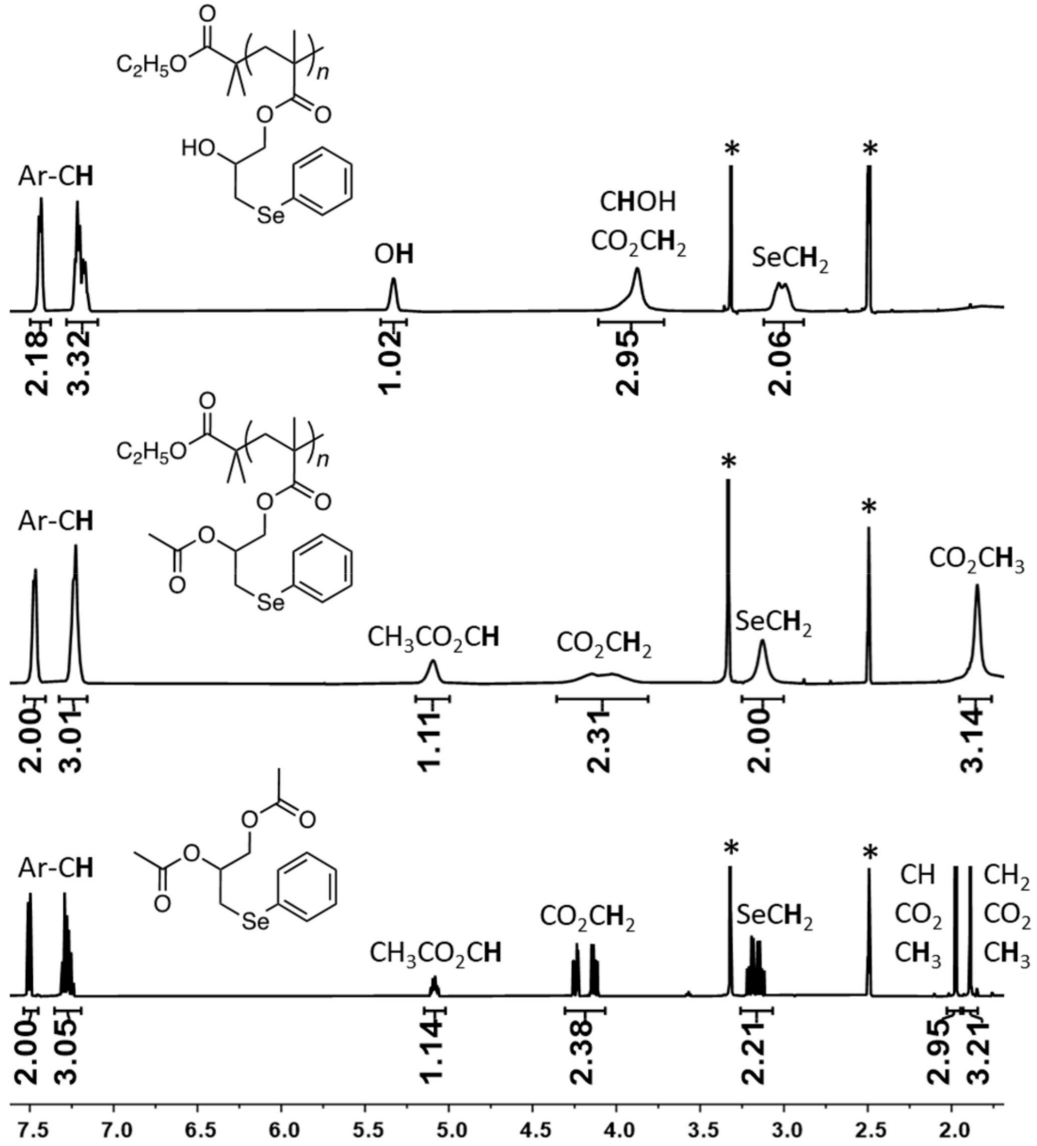
^1^H-NMR (DMSO-*d_6_*) of polymer **3** before (**top**) and after (**middle**) acetylation. The bottom shows a model compound of known regiochemistry prepared through selenium–halide reaction between bromopropane diol and benzeneselenol followed by acetylation [2]. The residual solvent signals from DMSO and water are shown with the help of an asterisk.

The regiochemistry of the ring-opening reaction could be verified by acetylation of the hydroxyl group (Figure 3) [1]. Upon acetylation, resonance from one proton shifted downfield to approximately 5 ppm. This indicated that only one proton was located adjacent to the hydroxyl group. It meant that the base-generated selenolate nucleophile attacked the least substituted carbon atom and produced a secondary hydroxyl group in the system. A lack of any other signals suggested that this isomer formed exclusively and the base-catalyzed ring-opening reaction was regiospecific.

**Scheme 2:**
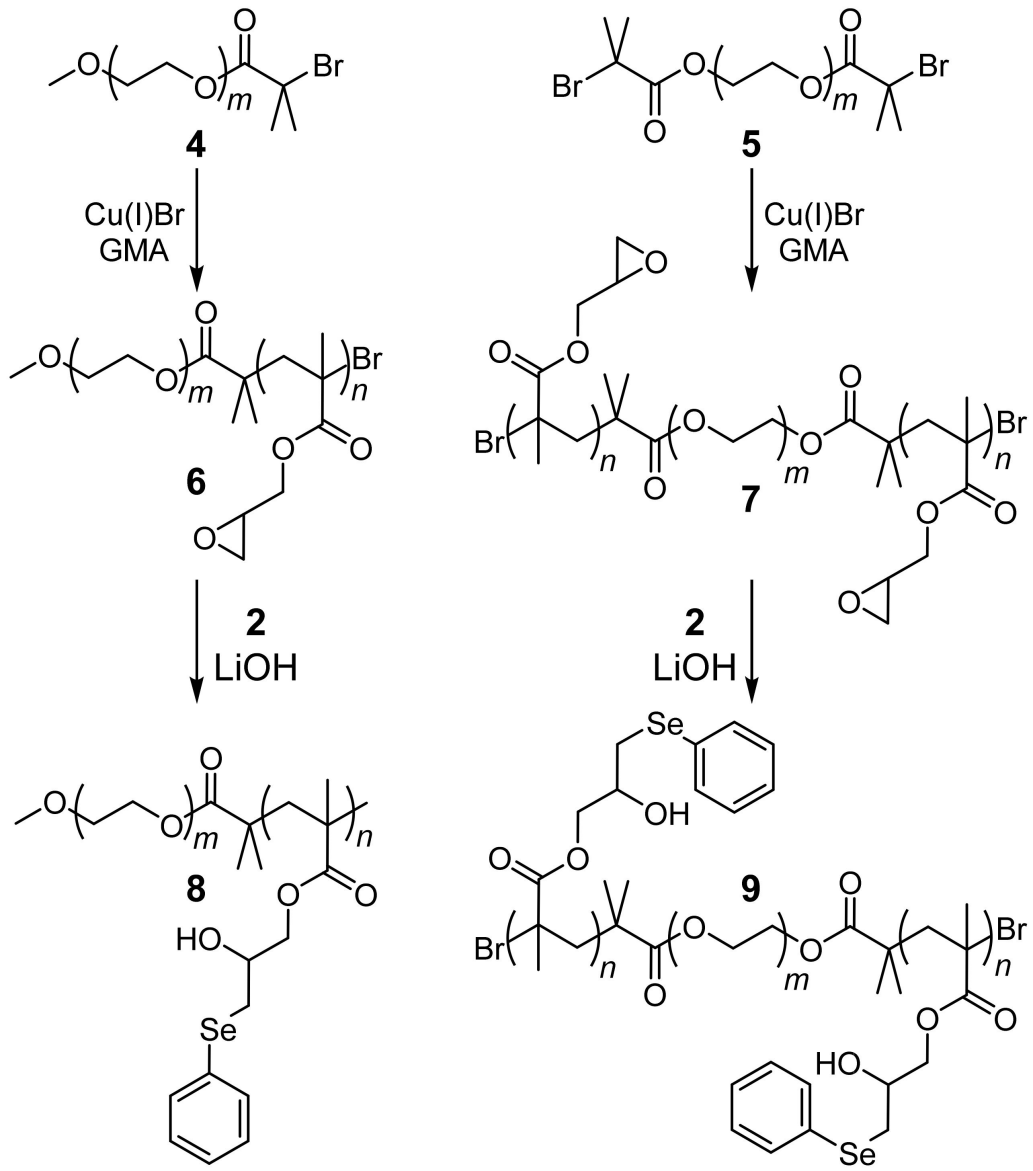
Synthesis of organoselenium block copolymers.

**Figure 4:**
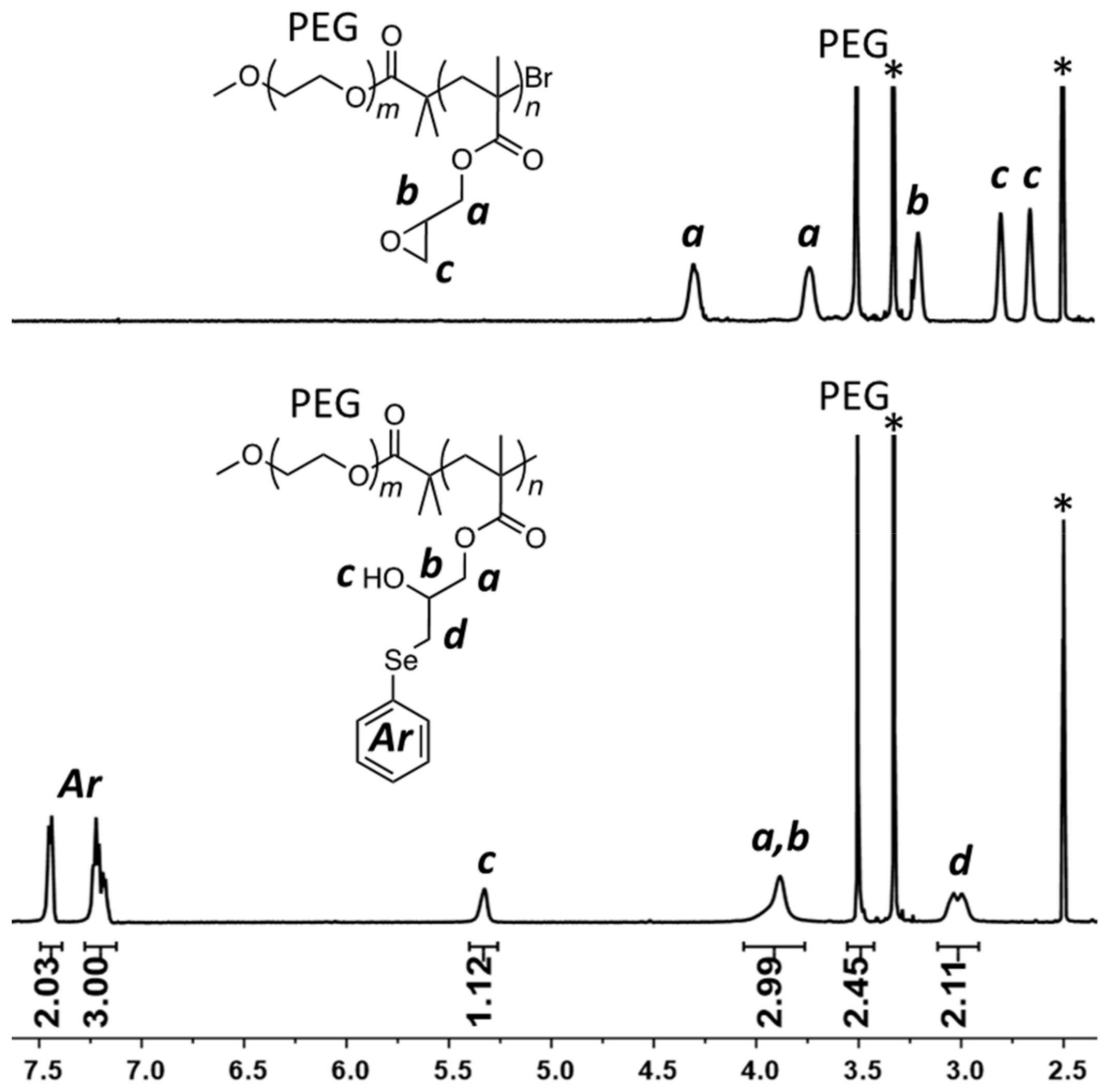
^1^H-NMR of **6** and **8** in DMSO-*d_6_*. The residual solvent signals from DMSO and water are shown with the help of an asterisk.

Encouraged by the optimization study, the synthesis of block copolymers was targeted [26,27]. For this, poly(ethylene glycol)-based macroinitiators **4** and **5** were chosen (Scheme 2). An atom transfer radical polymerization of these macroinitiators and glycidyl methacrylate monomer yielded AB (**6**: *M*_w(GPC)_ = 27,000, *M*_n(GPC)_ = 21,400, *M*_w_/*M*_n_ = 1.2) and ABA (**7**: *M*_w(GPC)_ = 37,500, *M*_n(GPC)_ = 29,100, *M*_w_/*M*_n_ = 1.2) block copolymers [28]. The methylene groups from the poly(ethylene glycol) backbone could be observed at 3.5 ppm in ^1^H-NMR spectra of the block copolymers (Figure 4 and Figure S9). An area integration analysis suggested that polymers **6** and **7** contained about 185 and 210 average number of glycidyl units, respectively. These groups could be functionalized with benzeneselenol to furnish organoselenium polymers **8** (*M*_w(GPC)_ = 36,100, *M*_n(GPC)_ = 28,000, *M*_w_/*M*_n_ = 1.2) and **9** (*M*_w(GPC)_ = 50600, *M*_n(GPC)_ = 38,300, *M*_w_/*M*_n_ = 1.3) under the optimized conditions. Once again, the functionalization reactions did not affect the polymer dispersity in an adverse manner, and a change in retention time to lower values is observed after the polymer functionalization process (Figures S10 and S11).

**Scheme 3:**
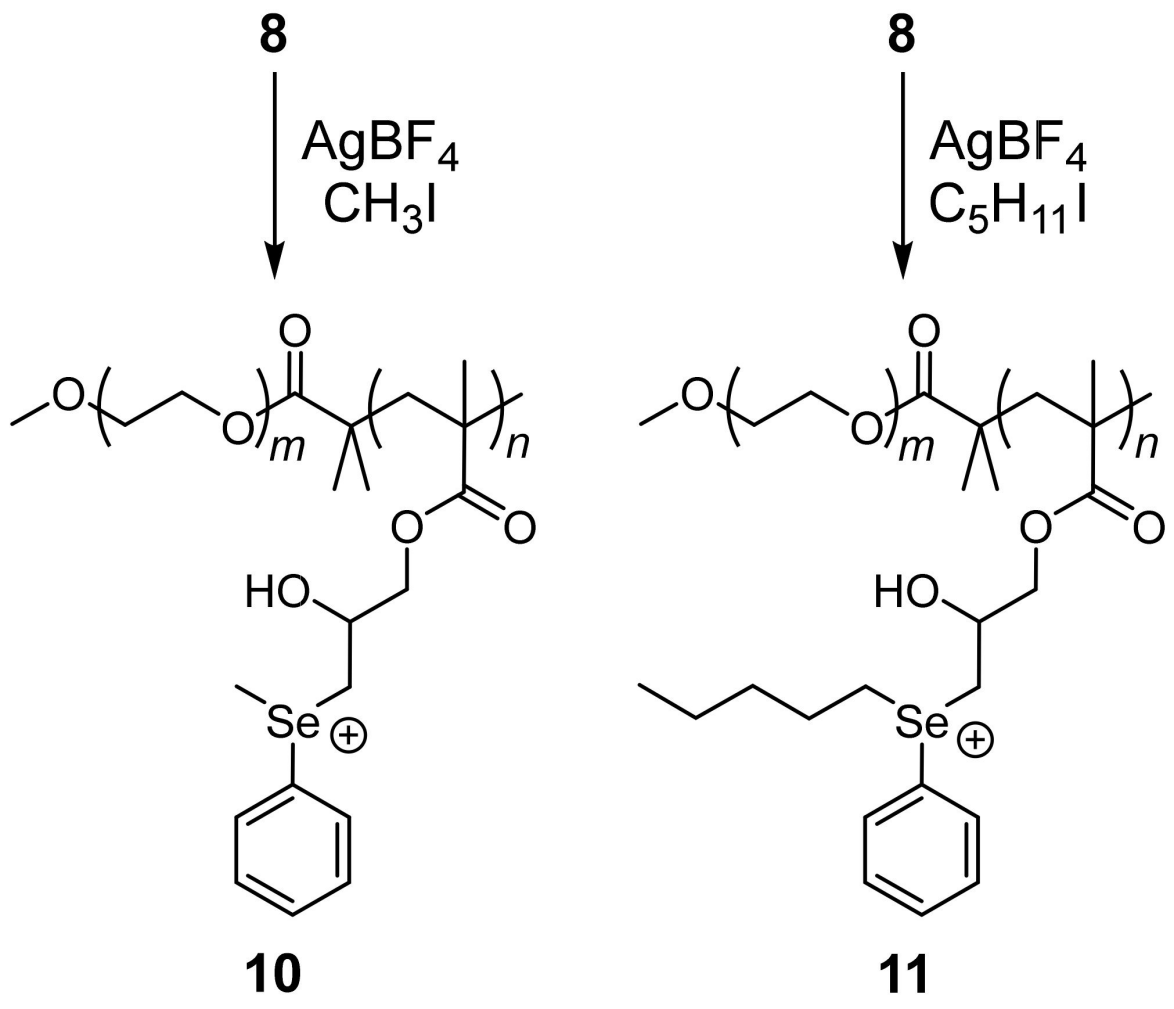
Alkylation of selenium atoms to access selenonium polymers.

**Figure 5:**
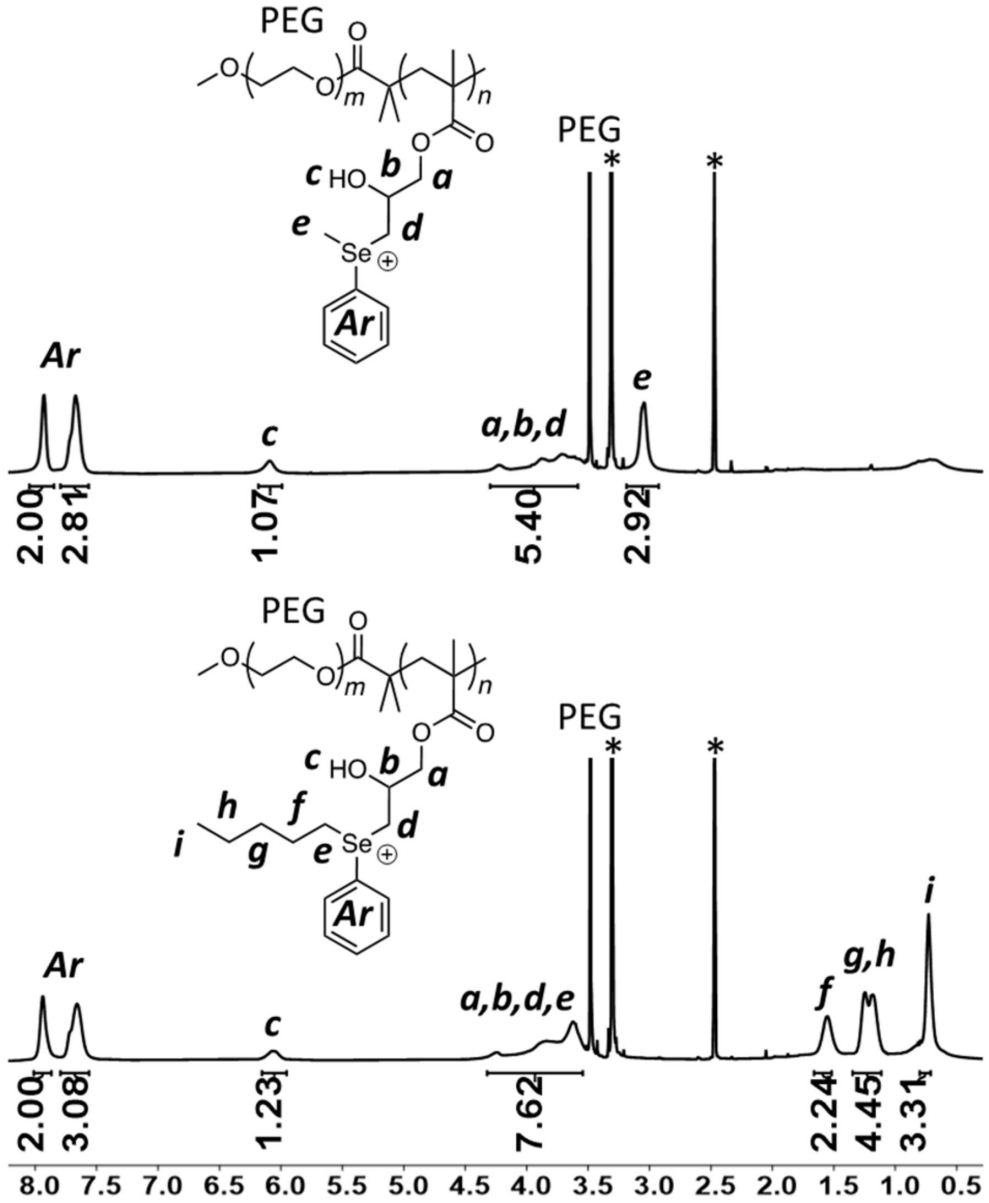
^1^H-NMR of **10** and **11** in DMSO-*d_6_*. The residual solvent signals from DMSO and water are shown with the help of an asterisk.

Next, alkylation of the side-chain selenium atoms was considered. For this, methyl iodide and pentyl iodide were chosen as the alkylating agents (Scheme 3). As can be seen in Figure 5, the transformation of selenium to an electron-deficient selenium cation results in a downfield shift of the aromatic signals and adjacent methylene protons. In the case of **10**, the newly introduced methyl group can be seen at 3 ppm, while in the case of **11**, the aliphatic chain could be observed in the range of 0.5–2 ppm. The area integration analyses in both cases indicate a complete ionization of the structures.

**Figure 6:**
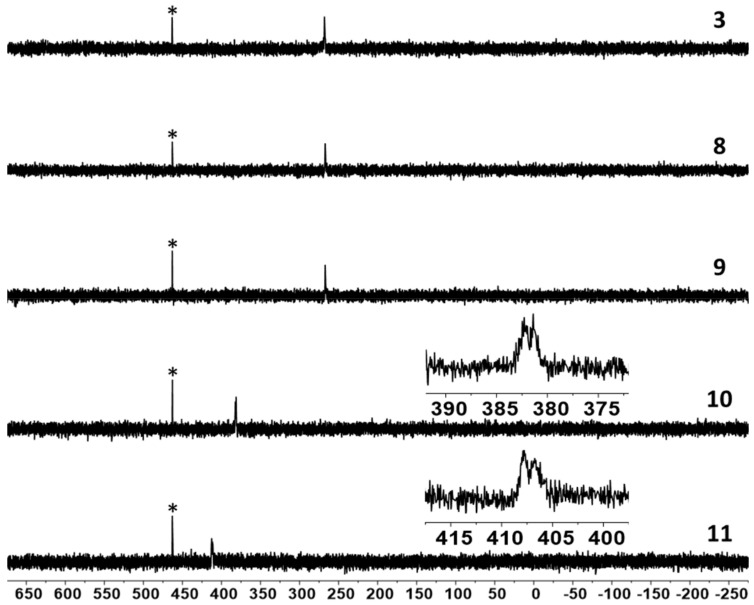
^77^Se-NMR of polymers **3**, **8**, **9**, **10**, and **11**. The signal from the standard (PhSeSePH) is shown with the help of an asterisk.

**Figure 7:**
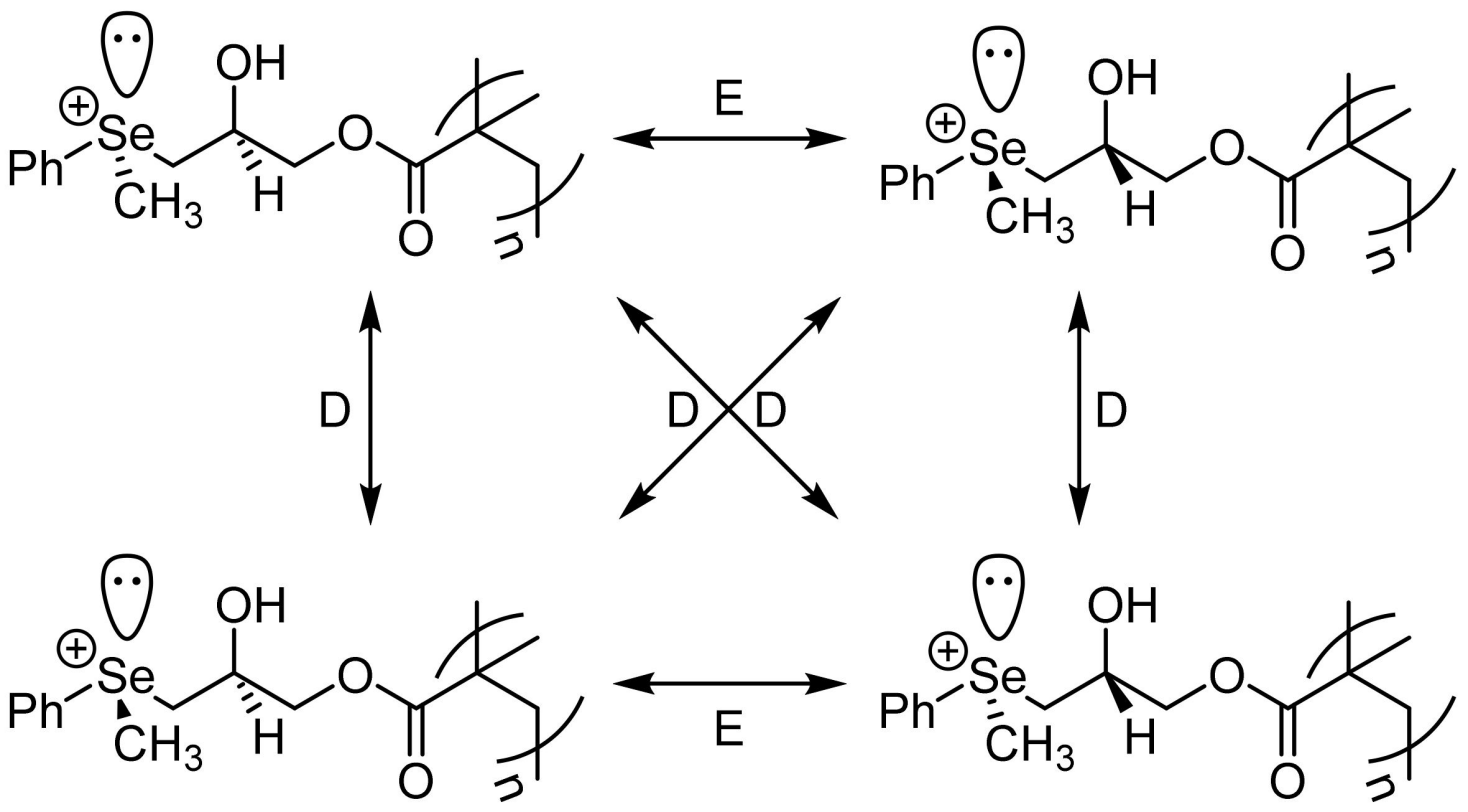
Stereochemical considerations upon alkylation of the ß-hydroxy selenide linkage in organoselenium polymers (S = stereoisomer, D = diastereomer).

Finally, the synthesized polymers were studied with the help of ^77^Se-NMR spectroscopy. In this analysis, diphenyldiselenide was used as a standard with a chemical shift of 463 ppm [29]. This study indicated that neutral selenium atoms in polymers **3**, **8**, and **9** resonate at 267 ppm (Figure 6). However, once the polymers become cationic, a large downfield shift (> 110 ppm) is observed due to the electron-poor nature of the cationic selenium species in polymers **10** and **11**. Furthermore, in the case of ionic structures, two signals could be observed due to the formation of diastereomers in the system (Figure 7).

## 3. Conclusions

In summary, base-catalyzed selenium-epoxy reaction is an efficient method to prepare organoselenium polymers. An optimization study on poly(glycidyl methacrylate) indicated that LiOH is the best catalyst as it can be used in chloroform as well as aqueous THF in 5.9% loadings to quantitatively open the side-chain epoxides in 3 h of reaction time. These conditions can be successfully applied to AB and ABA-type block copolymers containing a poly(ethylene glycol) segment. Finally, the selenium atoms can be alkylated to afford cationic polymers. In essence, this study establishes the use of selenol nucleophiles in the ring-opening reaction of polymeric glycidyl scaffolds and shows the generality of the selenonium polyelectrolyte structures by preparing (polyethylene glycol)-containing AB and ABA-types of block copolymers.

## Figures and Tables

**Table 1:** Details of the ring-opening reaction in polymer **1**.

Entry	Solvent	Selenol (2)	Catalyst	Catalyst (mol%) *^a^*	Catalyst (eq./SeH)	Time (hour)	Ring-Opening (%)
1	CHCl_3_	1.25	TEA	3.6	0.03	12	65
2	CHCl_3_	1.25	DBU	3.6	0.03	12	80
3	CHCl_3_	1.25	LiOH	3.6	0.03	12	84
4	THF	1.25	TEA	3.6	0.03	12	59
5	THF	1.25	DBU	3.6	0.03	12	74
6	THF/H_2_O	1.25	LiOH	3.6	0.03	12	>99
7	CHCl_3_	1.25	TEA	5.9	0.05	12	91
8	CHCl_3_	1.25	DBU	5.9	0.05	12	91
9	CHCl_3_	1.25	LiOH	5.9	0.05	12	>99
10	THF	1.25	TEA	5.9	0.05	12	91
11	THF	1.25	DBU	5.9	0.05	12	>99
12	THF/H_2_O	1.25	LiOH	5.9	0.05	12	>99
13	CHCl_3_	1.25	TEA	5.9	0.05	3	78
14	CHCl_3_	1.25	DBU	5.9	0.05	3	88
15	CHCl_3_	1.25	LiOH	5.9	0.05	3	>99
16	THF	1.25	TEA	5.9	0.05	3	83
17	THF	1.25	DBU	5.9	0.05	3	83
18	THF/H_2_O	1.25	LiOH	5.9	0.05	3	>99
19	CHCl_3_	1.25	TEA	5.9	0.05	1	64
20	CHCl_3_	1.25	DBU	5.9	0.05	1	68
21	CHCl_3_	1.25	LiOH	5.9	0.05	1	77
22	THF	1.25	TEA	5.9	0.05	1	55
23	THF	1.25	DBU	5.9	0.05	1	62
24	THF/H_2_O	1.25	LiOH	5.9	0.05	1	86

*^a^* Catalyst mol% = {mol of catalyst/(mol of epoxide unit + mol of catalyst)} × 100.

## Supplementary Materials

The following are available online at https://www.mdpi.com/2073-4360/12/11/2685/s1, Figure S1: ^1^H-NMR spectra for entries 1-3 in Table 1 (DMSO-*d_6_*), Figure S2: ^1^H-NMR spectra for entries 4-6 in Table 1 (DMSO-*d_6_*), Figure S3: ^1^H-NMR spectra for entries 7-9 in Table 1 (DMSO-*d_6_*), Figure S4: ^1^H-NMR spectra for entries 10-12 in Table 1 (DMSO-*d_6_*), Figure S5: ^1^H-NMR spectra for entries 13-15 in Table 1 (DMSO-*d_6_*), Figure S6: ^1^H-NMR spectra for entries 16-18 in Table 1 (DMSO-*d_6_*), Figure S7: ^1^H-NMR spectra for entries 19-21 in Table 1 (DMSO-*d_6_*), Figure S8: ^1^H-NMR spectra for entries 22-24 in Table 1 (DMSO-*d_6_*), Figure S9: ^1^H-NMR of **7** (top) and **9** (bottom) (DMSO-*d_6_*), Figure S10: GPC trace for **6** (line) and **8** (dash) (THF), Figure S11: GPC trace for **7** (line) and **9** (dash) (THF). References [1,28] are cited in the supplementary materials

## References

[1] Eom T., Khan A. (2020). Polyselenonium salts: Synthesis through sequential selenium-epoxy ‘click’ chemistry and Se-alkylation. Chem. Commun..

[2] Eom T., Khan A. (2020). Selenium-Epoxy ‘Click’ Reaction and Se-Alkylation—Efficient Access to Organo-Selenium and Selenonium Compounds. Chemistry.

[3] Tanini D., Capperucci A. (2019). Ring opening reactions of heterocycles with selenium and tellurium nucleophiles. New J. Chem..

[4] Pan X.Q., Driessen F., Zhu X.L., Du Prez F.E. (2017). Selenolactone as a Building Block toward Dynamic Diselenide-Containing Polymer Architectures with Controllable Topology. ACS Macro Lett..

[5] Chen S., Pan X., Zhu J., Zhu X. (2019). Synthesis of selenide-containing polymers by multicomponent polymerization based on γ-butyroselenolactone. Polym. Chem..

[6] Zhang C.J., Cao X.H., Zhang X.H. (2020). Metal-Free Alternating Copolymerization of Nonstrained gamma-Selenobutyrolactone with Epoxides for Selenium-Rich Polyesters. Macromolecules.

[7] Wang Y.N., Lin X., Zhang Z., Zhu J., Pan X., Zhu X. (2020). A Novel Synthesis of Poly (Ester-*Alt*-Selenide) s by Ring-Opening Copolymerization of γ-Selenobutyrolactone and Epoxy Monomer. Polymers.

[8] Lu W., An X., Gao F., Zhu J., Zhou N., Zhang Z., Pan X., Zhu X. (2017). Highly Efficient Chain End Derivatization of Selenol-Ended Polystyrenes by Nucleophilic Substitution Reactions. Macromol. Chem. Phys..

[9] Li Q., Zhang Y., Chen Z., Pan X., Zhang Z., Zhu J., Zhu X. (2020). Organoselenium chemistry-based polymer synthesis. Org. Chem. Front..

[10] Xia J., Li T., Lu C., Xu H. (2018). Selenium-Containing Polymers: Perspectives toward Diverse Applications in Both Adaptive and Biomedical Materials. Macromolecules.

[11] Ji S.B., Xia J.H., Xu H.P. (2016). Dynamic Chemistry of Selenium: Se-N and Se-Se Dynamic Covalent Bonds in Polymeric Systems. ACS Macro Lett..

[12] Xu H., Cao W., Zhang X. (2013). Selenium-Containing Polymers: Promising Biomaterials for Controlled Release and Enzyme Mimics. Accounts Chem. Res..

[13] Samal S.K., Dash M., Van Vlierberghe S., Kaplan D.L., Chiellini E., Van Blitterswijk C., Moroni L., Dubruel P. (2012). Cationic polymers and their therapeutic potential. Chem. Soc. Rev..

[14] Huber R., Criddle R. (1967). Comparison of the chemical properties of selenocysteine and selenocystine with their sulfur analogs. Arch. Biochem. Biophys..

[15] Tsarevsky N.V., Bencherif S.A., Matyjaszewski K. (2007). Graft Copolymers by a Combination of ATRP and Two Different Consecutive Click Reactions. Macromolecules.

[16] Jiang H., Pan X., Li N., Zhang Z., Zhu J., Zhu X. (2017). Selenide-containing high refractive index polymer material with adjustable refractive index and Abbe’s number. React. Funct. Polym..

[17] Theato P., Klok H.-A. (2013). Functional Polymers By Post-Polymerization Modification: Concepts, Guidelines and Applications.

[18] Günay K.A., Theato P., Klok H.A. (2013). Standing on the shoulders of hermann staudinger: Post-polymerization modification from past to present. J. Polym. Sci. Part A: Polym. Chem..

[19] Iha R.K., Wooley K.L., Nyström A.M., Burke D.J., Kade M.J., Hawker C.J. (2009). Applications of Orthogonal "Click" Chemistries in the Synthesis of Functional Soft Materials. Chem. Rev..

[20] Goldmann A.S., Glassner M., Inglis A.J., Barner-Kowollik C. (2013). Post-Functionalization of Polymers via Orthogonal Ligation Chemistry. Macromol. Rapid Commun..

[21] Stuparu M.C., Khan A. (2016). Thiol-epoxy “click” chemistry: Application in preparation and postpolymerization modification of polymers. J. Polym. Sci. Part A Polym. Chem..

[22] Muzammil E.M., Khan A., Stuparu M.C. (2017). Post-polymerization modification reactions of poly(glycidyl methacrylate)s. RSC Adv..

[23] Gadwal I., Stuparu M.C., Khan A. (2015). Homopolymer bifunctionalization through sequential thiol–epoxy and esterification reactions: An optimization, quantification, and structural elucidation study. Polym. Chem..

[24] De S., Khan A. (2012). Efficient synthesis of multifunctional polymers via thiol-epoxy “click” chemistry. Chem. Commun..

[25] Buerkli C., Lee S.H., Moroz E., Stuparu M.C., Leroux J.-C., Khan A. (2014). Amphipathic Homopolymers for siRNA Delivery: Probing Impact of Bifunctional Polymer Composition on Transfection. Biomacromolecules.

[26] Han P., Ma N., Ren H., Xu H., Li Z., Wang Z., Zhang X. (2010). Oxidation-Responsive Micelles Based on a Selenium-Containing Polymeric Superamphiphile. Langmuir.

[27] Ren H., Wu Y., Ma N., Xu H., Zhang X. (2012). Side-chain selenium-containing amphiphilic block copolymers: Redox-controlled self-assembly and disassembly. Soft Matter.

[28] De S., Stelzer C., Khan A. (2012). A general synthetic strategy to prepare poly(ethylene glycol)-based multifunctional copolymers. Polym. Chem..

[29] Duddeck H. (1995). Selenium-77 nuclear magnetic resonance spectroscopy. Prog. Nucl. Mag. Res. Spectrosc..

