# Supplementary material for: Selenonium Polyelectrolyte Synthesis through Post-Polymerization Modifications of Poly (Glycidyl Methacrylate) Scaffolds
Source: Polymers (Basel). 2020 Nov 13;12(11):2685. doi: 10.3390/polym12112685 (PMC7697662; doi:10.3390/polym12112685)
Supplement: Supplementary file 1 [file polymers-12-02685-s001.pdf]

Supplementary Materials

Selenonium Polyelectrolyte Synthesis through Post-Polymerization Modifications of Poly (glycidyl methacrylate) Scaffolds

Taejun Eom and Anzar Khan *

Department of Chemical and Biological Engineering, Korea University, 145 Anam-Ro, Seongbuk-Gu, Seoul 02841, Korea; eomtorr@korea.ac.kr

* Correspondence: anzar@korea.ac.kr; Tel.: +82-232-904-859

1. General Methods and Materials

Glycidyl methacrylate, ethyl α-bromoisobutyrate (EBiB) 4,4′-dinonyl-2,2′-dipyridyl (dNbpy), Cu(I)Br, benzeneselenol, lithium hydroxide (LiOH), 1,8,-diazabicyclo[5.4.0]undec-7-ene (DBU), triethylamine (TEA), iodomethane, iodopentane and silver tetrafluoroborate ($AgBF_4$) were purchased from commercial sources. Polyethylene glycol-based macroinitiators were prepared via esterification of commercially available hydroxyl-terminated polyethylene glycols using a procedure given in Ref. [1]. For acetylation of polymer **3**, a procedure given in Ref. [2] was followed. NMR spectra were recorded on a Varian NMR system 500 MHz spectrometer, using DMSO-d_6 as the solvents. Gel permeation chromatography (GPC) (against polystyrene standards) was carried out in THF (1 mL/min, 40 °C) using a Waters system (Waters 1515 pump, Water 2414 refractive 5 index detector) instrument with three styragel HR 0.5, HR 2, HR 4 columns.

2. Polymer synthesis

Synthesis of PGMA (**1**): glycidyl methacrylate (5 g, 35.17 mmol), 4,4′-dinonyl-2,2′-dipyridyl (dNbpy) (81 mg, 0.20 mmol), ethyl α-bromoisobutyrate (EBiB) (19 mg, 0.1 mmol), and anisole (10 mL) added to a schlenk tube and was purged by bubbling Ar for 30 min. Cu(I)Br (14 mg, 0.1 mmol) was added and Ar purging was continued for another 5 min. The reaction mixture was then stirred under inert atmosphere at room temperature for overnight. After this time, the reaction mixture was cooled to room temperature and precipitated into isopropanol, filtered, and passed through a small plug of silica gel using THF as an eluent. The organic solvent was reduced under low pressure and then precipitated into isopropanol. The obtained white powder was then dried under high vacuum conditions. ^1H NMR (500 MHz, DMSO-d_6) δ 4.30 (br s, COO**CH_2**), 3.74 (br s, COO**CH_2**), 3.21 (br s, CH_2**CH**CH_2O), 2.80 (br s, $COOCH_2CH$**CH_2**O), 2.66 (br s, $COOCH_2CH$**CH_2**O), 2.17 – 0.57 (br m, backbone). GPC(THF): M_n = 36400, M_w = 41900, PDI (M_w/M_n) = 1.15.

Synthesis of PEG-*b*-PGMA (**6**): PEG macroinitiator (5 kDa) (618 mg, 0.12 mmol), glycidyl methacrylate (3 g, 21.1 mmol), 4,4′-dinonyl-2,2′-dipyridyl (dNbpy) (100 mg, 0.25 mmol), and anisole (3 mL) added to a schlenk tube and was purged by bubbling Ar for 30 min. Cu(I)Br (17.7 mg, 0.12 mmol) was added and Ar purging was continued for another 5 min. The reaction mixture was then stirred under inert atmosphere at room temperature for 3 hrs. After this time, the reaction mixture was cooled to room temperature and precipitated into isopropanol, filtered, and passed through a small plug of silica gel using THF as an eluent. The organic solvent was reduced under low pressure and then precipitated into isopropanol. The obtained white powder was then dried under high vacuum conditions. ^1H NMR (500 MHz, DMSO-d_6) δ 4.28 (br s, COO**CH_2**), 3.74 (br s, COO**CH_2**), 3.51 (br s, **CH_2CH_2**O), 3.23 (s, O**CH_3**), 3.22 (br s, CH_2**CH**CH_2O), 2.80 (br s, $COOCH_2CH$**CH_2**O), 2.66 (br s, $COOCH_2CH$**CH_2**O), 2.20 – 0.66 (br m, backbone). GPC(THF): M_n = 21400, M_w = 27000, PDI (M_w/M_n) = 1.26.

Synthesis of PGMA-*b*-PEG-*b*-PGMA (**7**): PEG dimacroinitiator (10 kDa) (700 mg, 0.07 mmol), glycidyl methacrylate (2 g, 14 mmol), 4,4′-dinonyl-2,2′-dipyridyl (dNbpy) (57 mg, 0.14 mmol), and anisole (3 mL) added to a schlenk tube and was purged by bubbling Ar for 30 min. Cu(I)Br (10 mg, 0.07 mmol) was added and Ar purging was continued for another 5 min. The reaction mixture was then stirred under inert atmosphere at room temperature for 3 hrs. After this time, the reaction mixture was cooled to room temperature and precipitated into isopropanol, filtered, and passed through a small plug of silica gel using THF as an eluent. The organic solvent was reduced under low pressure and then precipitated into isopropanol. The obtained white powder was then dried under high vacuum conditions. ^{1}H NMR (500 MHz, DMSO-d_6) δ 4.30 (br s, COO**CH$_2$**), 3.74 (br s, COO**CH$_2$**), 3.51 (br s, **CH$_2$CH$_2$**O), 3.21 (br s, CH$_2$**CH**CH$_2$O), 2.80 (br s, COOCH$_2$CH**CH$_2$**O), 2.66 (br s, COOCH$_2$CH**CH$_2$**O), 2.20 – 0.55 (br m, backbone). GPC(THF): M_n = 29100, M_w = 37500, PDI (M_w/M_n) = 1.29.

2.1. Selenium-epoxy reaction

Using triethylamine (TEA): To a solution of polymer **1** (30 mg, 0.211 mmol of epoxide unit) and benzeneselenol (41 mg, 1.25 eq. per epoxy unit) in 1 mL THF or $CHCl_3$ was added TEA (0.03 or 0.05 eq. per SeH unit) at 0 °C. The cooling was removed, and the reaction mixture was stirred at room temperature for 1, 3, or 12 hours. After this time, the reaction mixture was precipitated into hexane thrice and then dried under high vacuum conditions.

Using diazabicycloundecene (DBU): To a solution of polymer **1** (30 mg, 0.211 mmol of epoxide unit) and benzeneselenol (41 mg, 1.25 eq. per. epoxy unit) in 1 mL of THF or $CHCl_3$ was added DBU (0.03 or 0.05 eq. per SeH unit) at 0 °C. The cooling was removed, and the reaction mixture was stirred at room temperature for 1, 3, or 12 hours. After this time, the reaction mixture was precipitated into hexane thrice and then dried under high vacuum conditions.

Using LiOH (in aqueous THF): To a solution of polymer **1** (30 mg, 0.211 mmol of epoxide unit) and benzeneselenol (41 mg, 1.25 eq. per epoxy unit) in THF (0.9 mL) was added LiOH (0.03 or 0.05 eq. per SeH unit) in water (0.1 mL) at 0 °C. The cooling was removed, and the reaction mixture was stirred at room temperature for 1, 3, or 12 hours. After this time, the reaction mixture was diluted with DCM and washed with water. The organic layer was dried over sodium sulfate, reduced under low pressure, precipitated into hexane thrice, and then dried under high vacuum conditions.

Using LiOH (in $CHCl_3$): To a solution of polymer **1** (30 mg, 0.211 mmol of epoxide unit) and benzeneselenol (41 mg, 1.25 eq. per epoxy unit) in $CHCl_3$ (1 mL) was added LiOH (0.03 or 0.05 eq. per SeH unit) at 0 °C. The cooling was removed, and the reaction mixture was stirred at room temperature for 1, 3, or 12 hours. After this time, the cloudy reaction mixture was precipitated into hexane (using a syringe filter) thrice and then dried under high vacuum conditions.

3: ^{1}H-NMR (500 MHz, DMSO-d_6) δ 7.45 (br s, -Se**Ph**), 7.21 (br s, -Se**Ph**), 5.34 (br s, CH(**OH**)CH$_2$Se), 3.88 (br m, COO**CH$_2$CH**(OH)), 3.02 (br d, CH(OH)**CH$_2$**Se), 2.17 – 0.57 (br m, backbone). ^{77}Se-NMR (95 MHz, DMSO-d_6) δ 267.9. GPC(THF): M_n = 42500, M_w = 52400, PDI (M_w/M_n) = 1.23.

8: ^{1}H-NMR (500 MHz, DMSO-d_6) δ 7.45 (br s, -Se**Ph**), 7.21 (br s, -Se**Ph**), 5.33 (CH(**OH**)CH$_2$Se), 3.89 (br m, COO**CH$_2$CH**(OH)), 3.51 (br s, **CH$_2$CH$_2$**O), 3.23 (s, O**CH$_3$**), 3.02 (br d, CH(OH)**CH$_2$**Se), 2.20 – 0.66 (br m, backbone). ^{77}Se-NMR (95 MHz, DMSO-d_6) δ 267.1. GPC(THF): M_n = 28000, M_w = 36100, PDI (M_w/M_n) = 1.29.

9: ^{1}H-NMR (500 MHz, DMSO-d_6) δ 7.45 (br s, -Se**Ph**), 7.21 (br s, -Se**Ph**), 5.34 (CH(**OH**)CH$_2$Se), 3.89 (br m, COO**CH$_2$CH**(OH)), 3.51 (br s, **CH$_2$CH$_2$**O), 3.02 (br d, CH(OH)**CH$_2$**Se), 2.20 – 0.55 (br m, backbone). ^{77}Se-NMR (95 MHz, DMSO-d_6) δ 267.1. GPC(THF): M_n = 38300, M_w = 50600, PDI (M_w/M_n) = 1.32.

2.2. Se-Alkylation

To a solution of polymer **8** (10 mg) and $AgBF_4$ (1.25 eq. per seleno-ether unit) in 1 mL MeCN was added alkyl iodide (4 eq. per seleno-ether unit) and the reaction mixture was stirred under an inert atmosphere in the dark at 50 °C for 24 hours. After this time, the precipitated AgI was removed by syringe filter and the filtrate was extracted with hexane thrice [*ACS Omega.* **2020,** *5,* 13384-13388]. The MeCN layer was precipitated into diethyl ether thrice. The precipitated polymer was dissolved in MeCN and transferred to a dialysis tube (cutoff 1 kDa), and then dialyzed against DI water for 6 hours. Subsequently, the polymer solution was lyophilized to dryness.

10: ^{1}H-NMR (500 MHz, DMSO-d_6) δ 7.90 (br s, -Se^+**Ph**), 7.68 (br s, -Se^+**Ph**), 6.07 (br s, CH(**OH**)CH_2Se), 4.39 – 3.55 (br m, COO**CH$_2$CH**(OH)**CH$_2$**Se), 3.51 (br s, **CH$_2$CH$_2$**O), 3.23 (s, O**CH$_3$**), 3.07 (br d, -Se^+(**CH$_3$**)), 2.17 – 0.57 (br m, backbone). ^{77}Se-NMR (95 MHz, DMSO-d_6) δ 382.2, 381.3.

11: ^{1}H-NMR (500 MHz, DMSO-d_6) δ 7.94 (br s, -Se**Ph**), 7.67 (br s, -Se**Ph**), 6.07 (br s, CH(**OH**)CH_2Se), 4.38 – 3.58 (br m, **CH$_2$CH**(OH)**CH$_2$**Se^+(**CH$_2$**$CH_2CH_2CH_2CH_3$)), 3.51 (br s, **CH$_2$CH$_2$**O), 3.23 (s, O**CH$_3$**), 1.60 (br s, Se^+(CH_2**CH$_2$**$CH_2CH_2CH_3$)), 1.28 (br s, Se^+(CH_2CH_2**CH$_2$CH$_2$**CH_3)), 0.78 (br s, Se^+($CH_2CH_2CH_2CH_2$**CH$_3$**)), 2.19 – 0.56 (br m, backbone). ^{77}Se-NMR (95 MHz, DMSO-d_6) δ 407.7, 406.7.

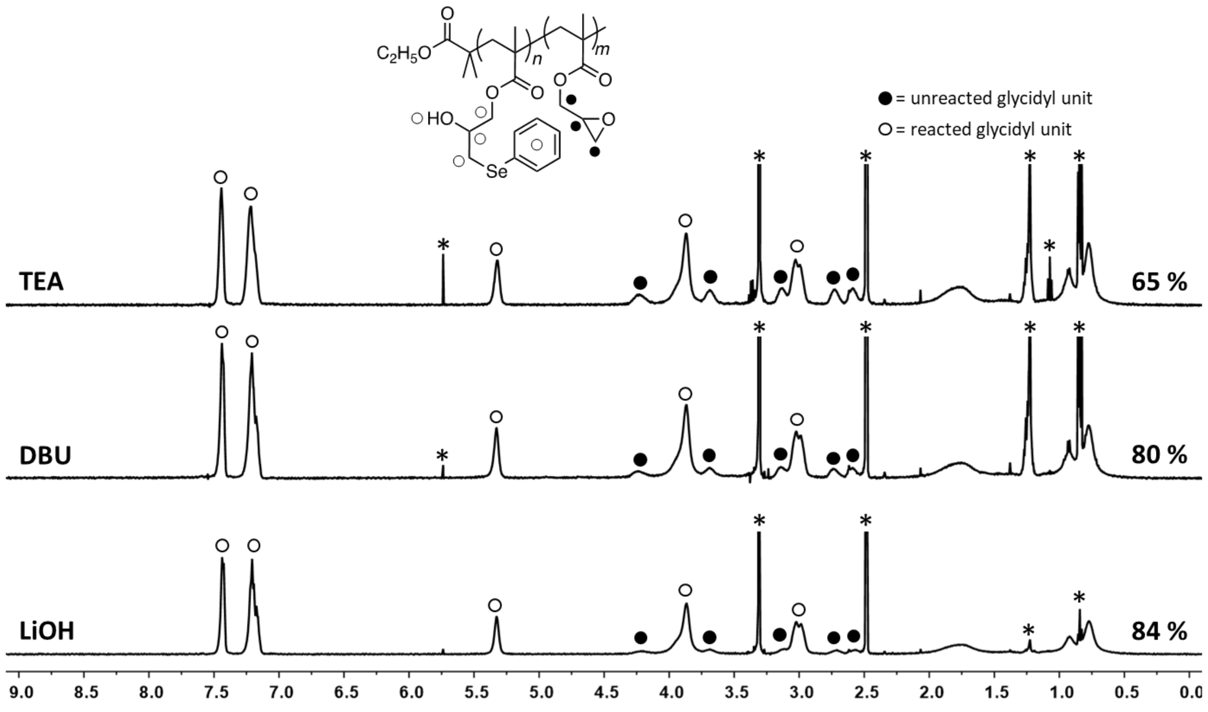

Figure S1. ^{1}H-NMR spectra for entries 1-3 in Table 1 (DMSO-d_6).

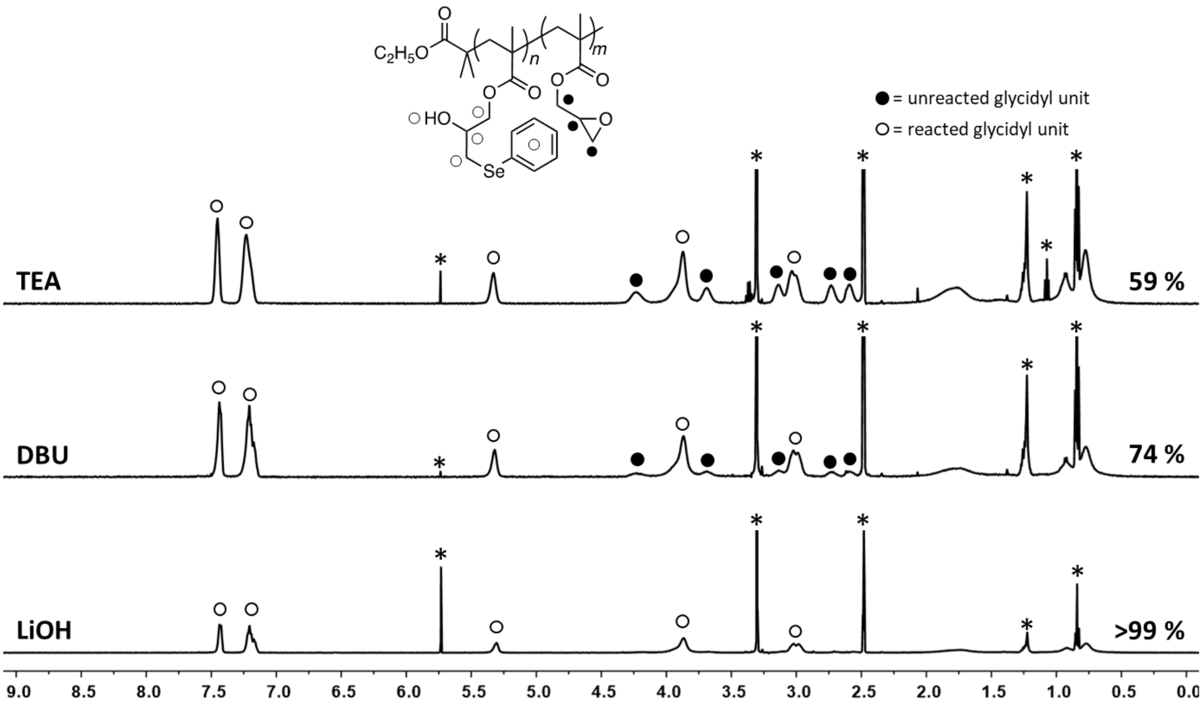

Figure S2. ^{1}H-NMR spectra for entries 4-6 in Table 1 (DMSO-d_6).

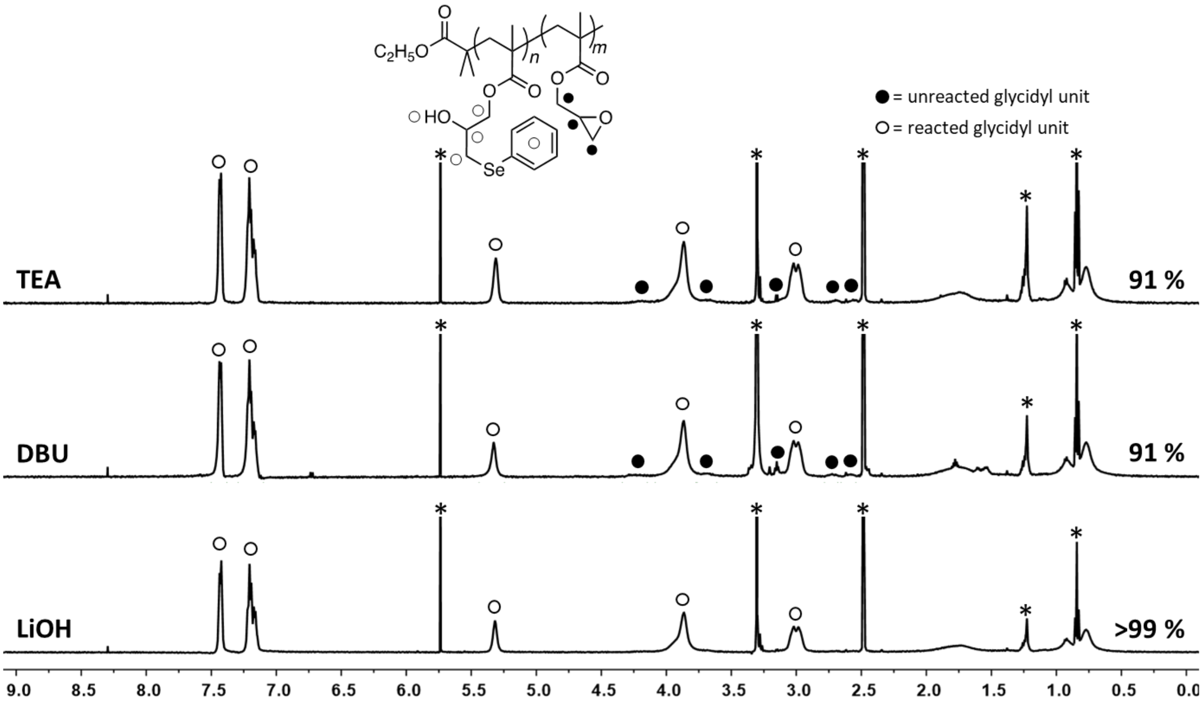

Figure S3. ^{1}H-NMR spectra for entries 7-9 in Table 1 (DMSO-d_6).

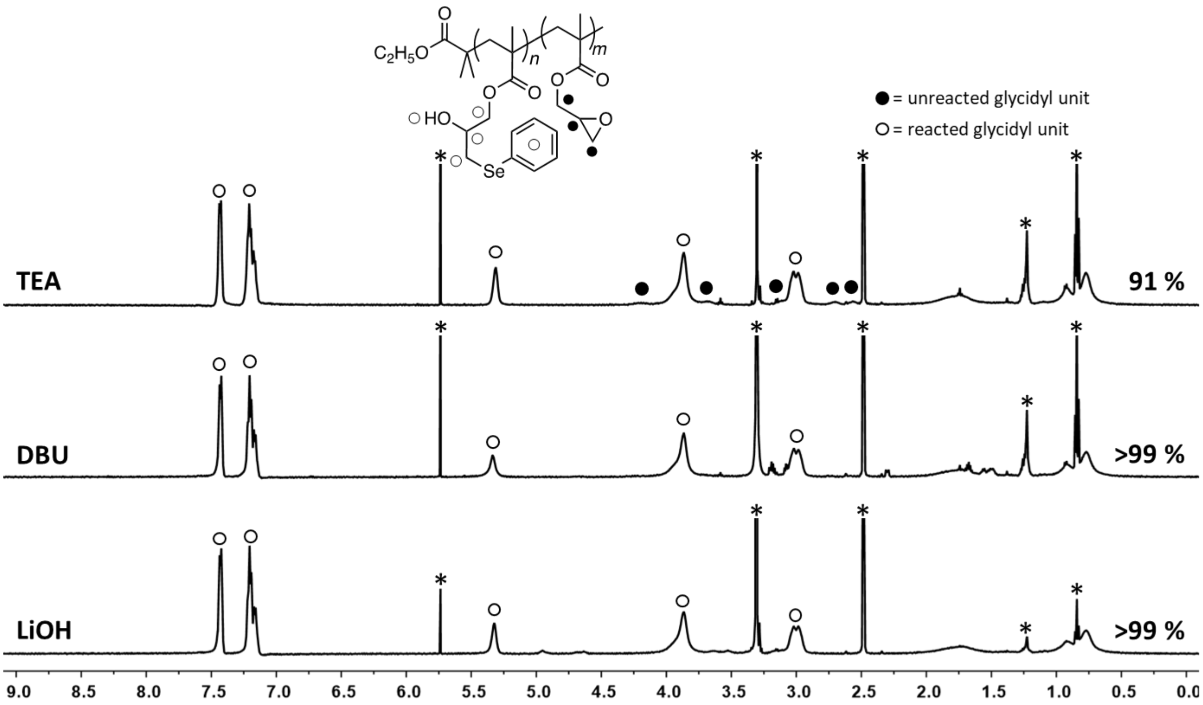

Figure S4. ^{1}H-NMR spectra for entries 10-12 in Table 1 (DMSO-d_6).

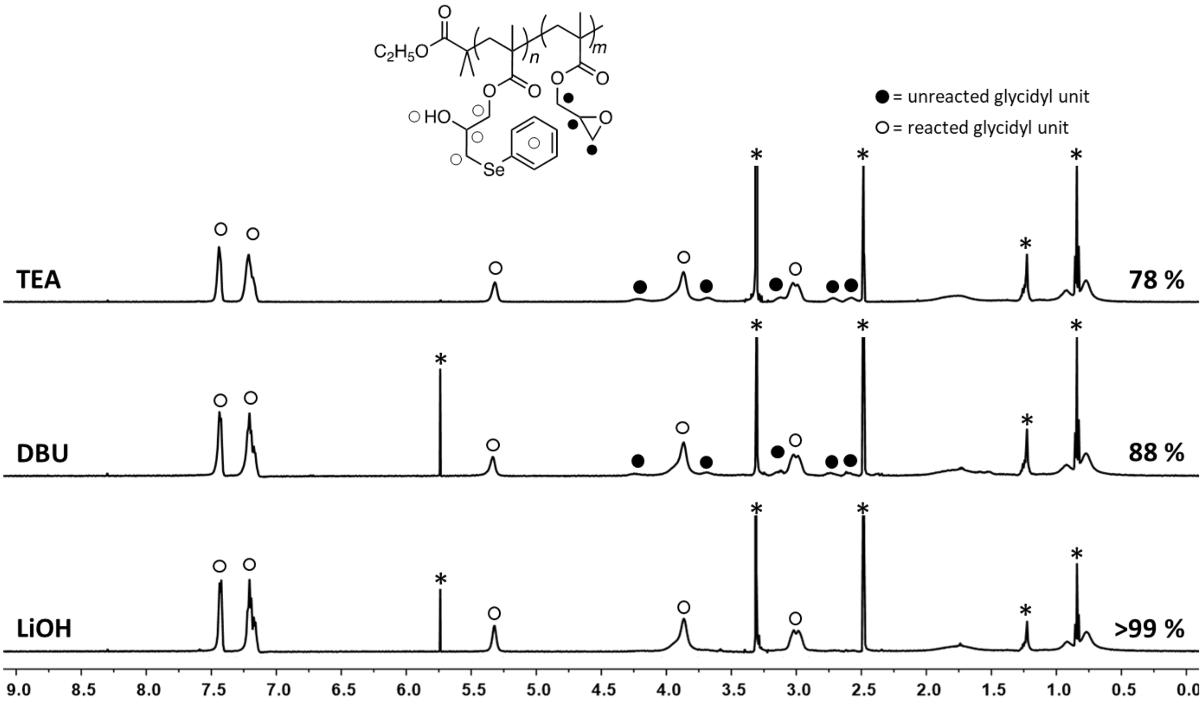

Figure S5. ^{1}H-NMR spectra for entries 13-15 in Table 1 (DMSO-d_6).

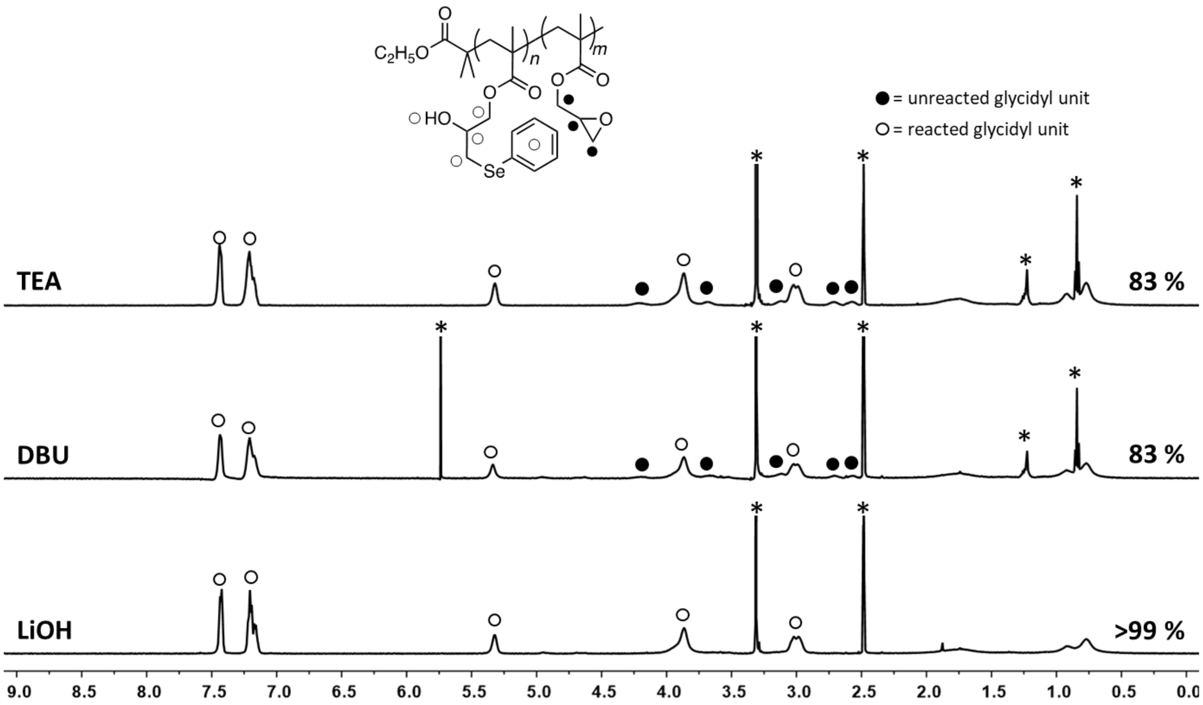

Figure S6. ^{1}H-NMR spectra for entries 16-18 in Table 1 (DMSO-d_6).

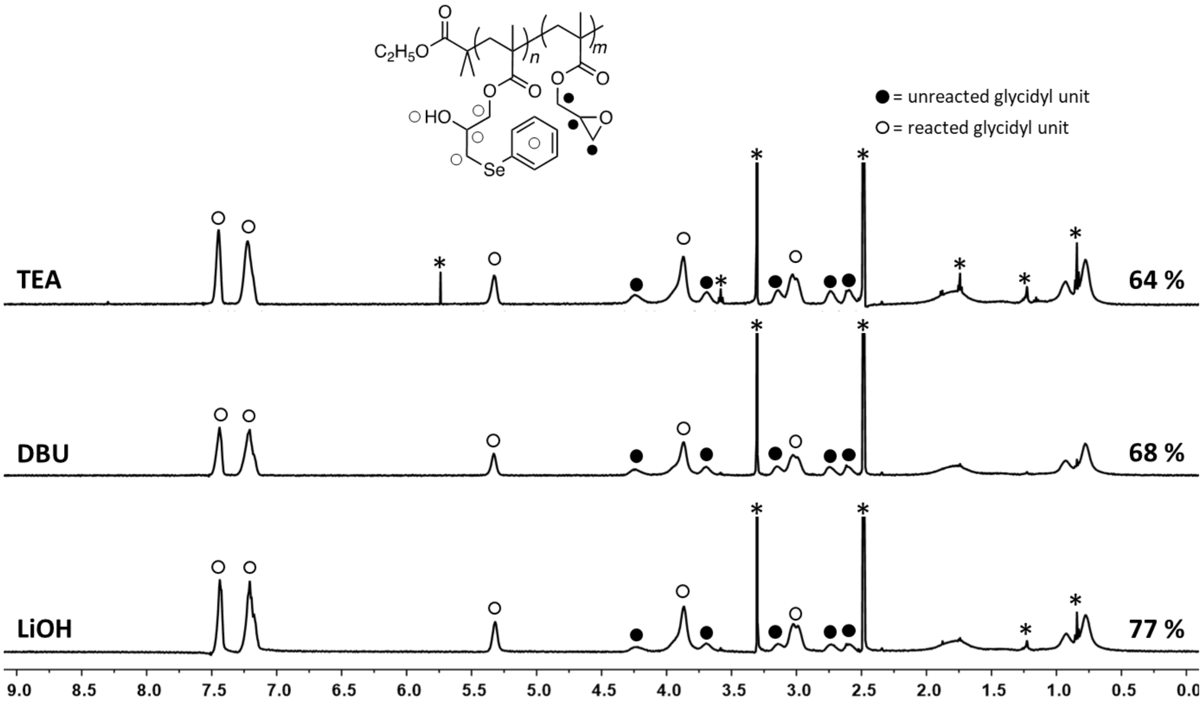

Figure S7. ^{1}H-NMR spectra for entries 19-21 in Table 1 (DMSO-d_6).

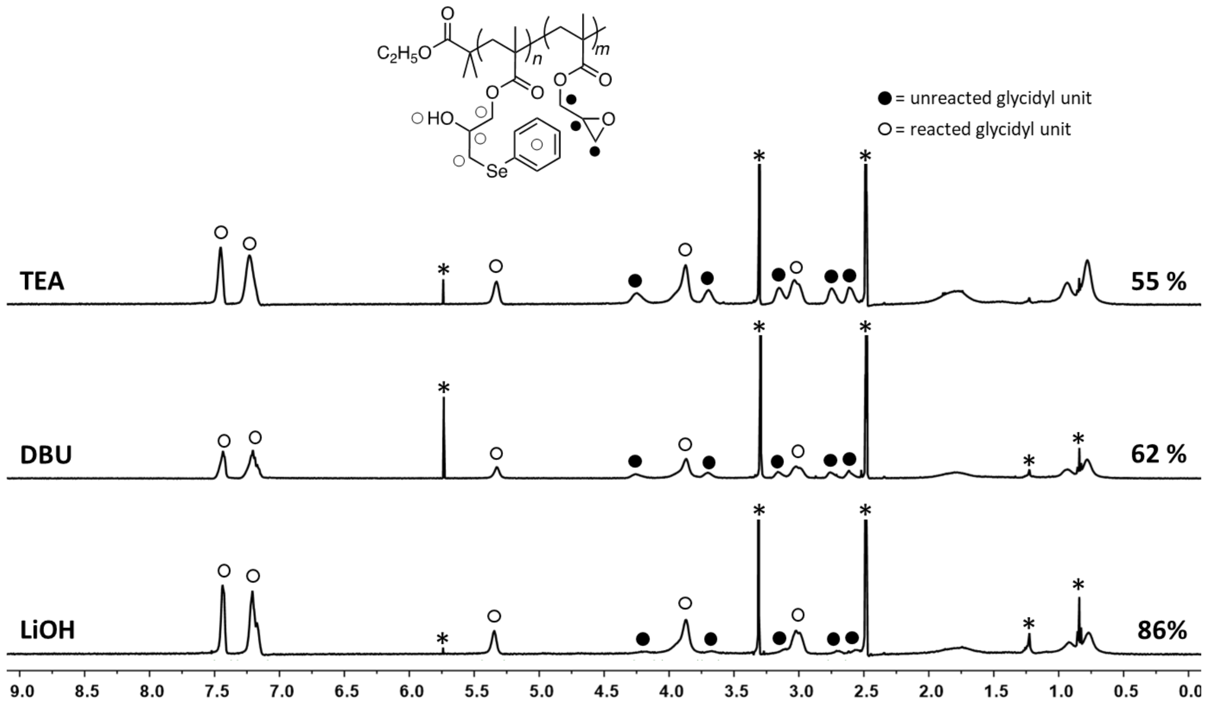

Figure S8. ^{1}H-NMR spectra for entries 22-24 in Table 1 (DMSO-d_6).

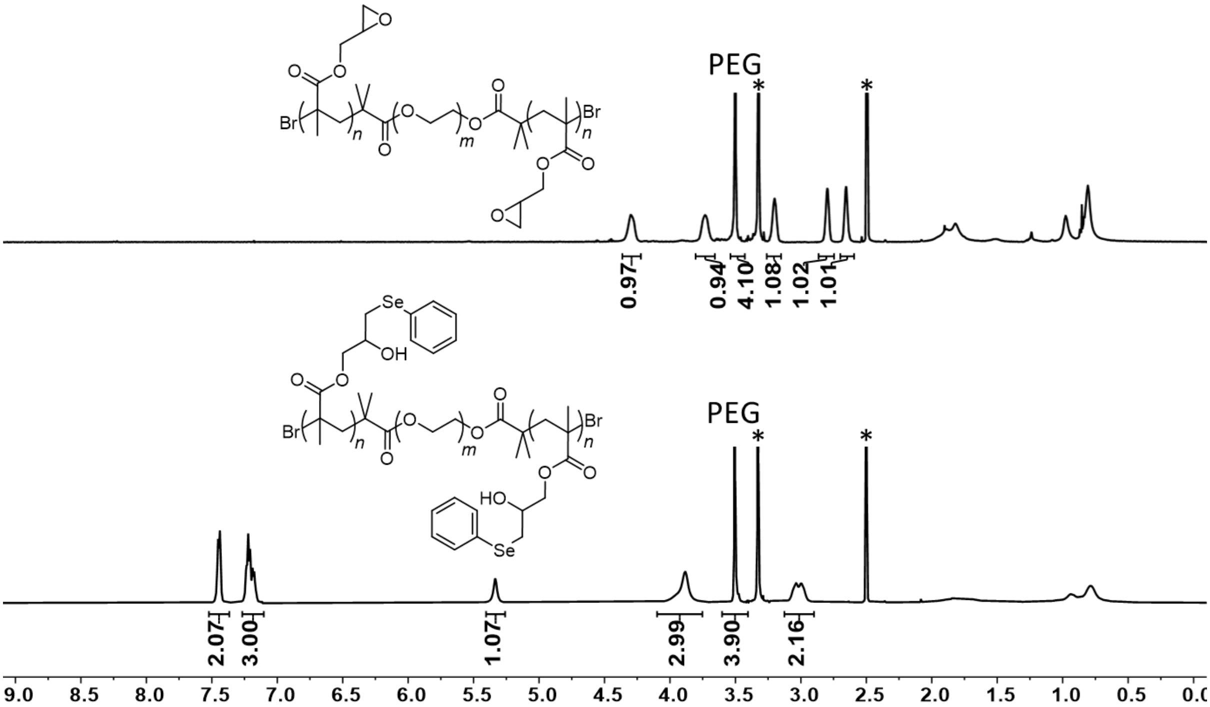

Figure S9. ^{1}H-NMR of **7** (top) and **9** (bottom) (DMSO-d_6).

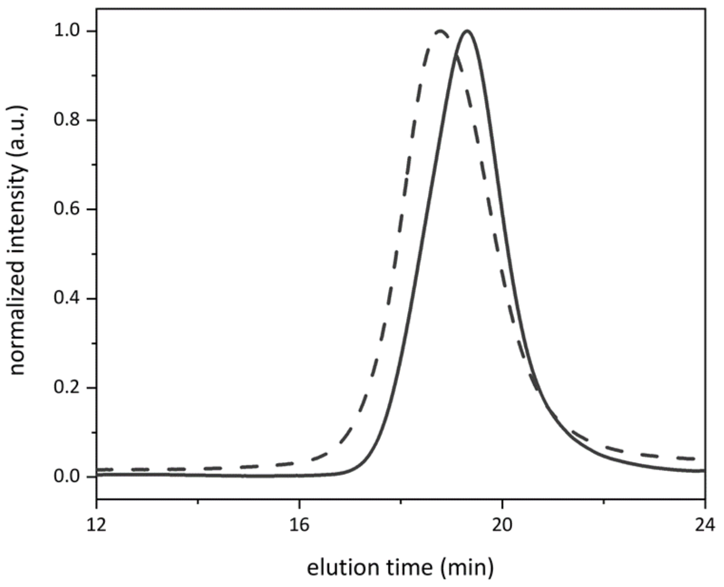

Figure S10. GPC trace for **6** (line) and **8** (dash) (THF).

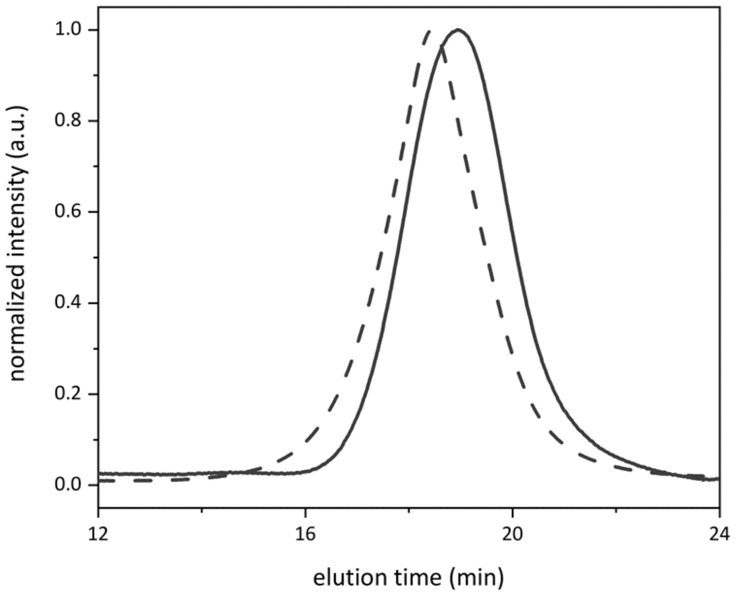

Figure S11. GPC trace for **7** (line) and **9** (dash) (THF).

References

1. De, S.; Stelzer, C.; Khan, A. A general synthetic strategy to prepare poly(ethylene glycol)-based multifunctional copolymers. *Polym. Chem.* **2012**, *3*, 2342–2345.
2. Eom, T.; Khan, A. Polyselenonium salts: Synthesis through sequential selenium-epoxy 'click' chemistry and Se-alkylation. *Chem. Commun.* **2020,** DOI: 10.1039/D0CC06653B.
